# Developing genetic counselling services in an underdeveloped healthcare setting

**DOI:** 10.1007/s12687-021-00546-z

**Published:** 2021-09-20

**Authors:** Andrada Ciucă, Ramona Moldovan, Adriana Băban

**Affiliations:** 1 Department of Psychology, Babeş-Bolyai University, Cluj-Napoca, Romania; 2 Division of Evolution and Genomic Sciences, School of Biological Science, University of Manchester, Manchester, UK; 3 Manchester Centre for Genomic Medicine, St Mary’s Hospital, Manchester University Hospitals NHS Foundation Trust, Manchester Academic Health Science Centre, Manchester, UK

**Keywords:** Cancer genetic counselling, Health communication, Service delivery

## Abstract

Genetic counselling services are well established in North America, Western Europe and Australia. In other regions, genetic counselling services are still emerging. Where this is the case, an in-depth understanding of the main stakeholders’ needs, challenges and opportunities will inform the changes and innovations required to bring genetic counselling closer to the community. The present study explored the needs and challenges of patients, family members and professionals with a view to setting up a cancer genetic counselling service in Romania. In order to get a comprehensive outlook, key stakeholders were interviewed using data source triangulation method. Thirty-four semi-structured interviews were conducted (13 patients, 11 family members and 10 professionals). Thematic analysis was used to explore and identify needs, barriers and opportunities in emerging cancer genetic counselling services. Three major themes were identified: (1) the “Needs” theme mainly focuses on various types of support that participants mentioned wanting: psychosocial, peer and additional support; (2) the “Challenges” theme includes aspects related to limited access to healthcare, lack of integrated services and pressure on the families; (3) the “Hopes” theme highlights the wish for integrated healthcare and an empathic rapport with healthcare providers. Our findings highlighted the main needs, challenges and hopes the patients, family members and professionals have and provides the groundwork for setting up cancer genetic counselling services.

## Introduction

Genetic counselling is aimed at “helping people understand and adapt to the medical, psychological and familial implications of genetic contributions to disease. This process integrates the following: (1) interpretation of family and medical history to assess the chance of disease occurrence or recurrence; (2) education about inheritance, testing, management, prevention, resources and research; (3) counselling to promote informed choices and adaptation to the risk or condition” (Resta et al., 2006). Genetic counselling has become an established service in North America, Western Europe and Australia and we now have substantial empirical evidence supporting its efficacy (Braithwaite, Emery, Walter, Prevost, & Sutton, 2004, Meiser & Halliday, 2002; Smerecnik, Mesters, Verweij, de Vries, & de Vries, 2009). Although access to genetic counselling remains uneven globally, there are ongoing efforts and improvements across standards of practice, training and regulation (Abacan et al., 2019).

In cancer settings, genetic counselling aims to identify and provide support to individuals affected by/at increased risk of an inherited predisposition to cancer. Cancer genetic counselling usually includes (1) taking a detailed personal and familial medical history, (2) assessment of genetic cancer risk, (3) facilitating informed consent for genetic testing, (4) disclosure of genetic test results and (5) psychosocial assessment (Riley et al., 2012). Cancer genetic counselling often, but not always, includes genetic testing. State-of-the art guidelines (e.g., National Society of Genetic Counselors and American Society of Clinical Oncology) recommend testing when certain conditions are met. These include (1) clinical conditions (e.g., a suggestive family history for inherited cancer; the test has an influence on medical management for individuals or the family) and (2) ethical conditions (e.g., testing is voluntary and informed consent is given; benefits of the test outweigh the risks; and test results can be adequately interpreted) (Riley et al., 2012; Robson, Storm, Weitzel, Wollins, & Offit, 2010).

Two systematic reviews have shown that cancer genetic counselling improves a large variety of outcomes (Athens et al., 2017; Madlensky et al., 2017). These include cognitive outcomes (e.g., knowledge, perceived personal control, risk perception accuracy and decisional conflict), affective outcomes (e.g., anxiety, cancer-related worry, psychological wellbeing), behavioural outcomes (e.g., positive health behaviours, uptake of genetic testing or screening, medical management), and other outcomes such as satisfaction and sharing information (Athens et al., 2017; Madlensky et al., 2017).

Patients in Romania, as in many other countries, have limited access to integrated healthcare services and there is a disconnection between highly specialized care and primary or community care (Vlădescu et al., 2016). There is an increasing trend to provide integrated and personalized healthcare services, however there are several systemic challenges that need to be addressed. In cancer settings, limited funds are available for reimbursement of specialized services such as genetic testing, and the National Health Insurance House is planning to improve access to genetic testing in oncology setting (National Health Insurance House, 2019). National screening programs for cancer are starting to develop, with ongoing or pilot programs, but there are no national screening programs for several types of cancer, including colorectal cancer (Cancer screening in the European Union, 2017). Furthermore, national guidelines and professional recommendations for psychosocial care in cancer settings are essentially lacking (Dégi, 2016). Genetic counselling is not yet recognized as a distinct healthcare profession even though there are approximately 75 trained genetic counsellors in the country (Abacan et al. 2019).

Our main objective was to gain an in-depth perspective of the needs, barriers and opportunities in the development of cancer genetic counselling services with input from patients, family members and professionals working in cancer settings.

## Method

### Participants

We conducted 34 semi-structured interviews using data source triangulation method (Carter et al., 2014) to collect a comprehensive set of diverse experiences and to ensure data saturation; we interviewed 13 patients, 11 family members and 10 healthcare professionals. Purposive sampling was used to recruit participants based on their potential need to access (i.e., patients and family members) or to recommend genetic counselling (i.e., professionals with diverse backgrounds such as genetic counselling, genetics, oncology, surgery, psychology or social work). Participants were recruited from the Oncology Institute in Cluj-Napoca, Romania, and several affected individuals from cancer patients’ associations. Participants’ characteristics are summarized in Tables 1, 2 and 3.

**Table 1:** Patients’ characteristics

Patient	Gender	Age	Diagnosis	Years since diagnosis
1	F	64	Chronic myeloid leukemia	10
2	F	38	Chronic myeloid leukemia	14
3	F	50	Chronic myeloid leukemia	13
4	F	72	Breast cancer	2
5	F	64	Breast cancer	10
6	F	52	Breast cancer	1
7	F	56	Breast cancer	1
8	F	52	Breast cancer	1
9	F	49	Colon cancer	3
10	M	43	Testicular cancer	1
11	M	40	Osteosarcoma	1
12	F	61	Breast cancer	1
13	F	63	Breast cancer	1

**Table 2:** Family members’ characteristics

Family member	Gender	Age	Affected family member	Diagnosis
1	F	32	Parent	Kidney cancer
2	F	28	Parent	Colon cancer
3	F	28	Aunt	Endometrial cancer
4	F	52	Parent	Eye cancer
5	M	63	Parent	Prostate cancer
6	F	49	Sister	Breast cancer
7	M	45	Partner	Neck cancer
8	F	44	Parent	Prostate cancer
9	F	29	Parent	Ovarian cancer
10	M	50	Partner	Breast cancer
11	F	25	Parent	Breast and uterus cancer

**Table 3:** Professionals’ characteristics

Professional	Gender	Age	Specialty	Years of experience
1	F	25	Lab biology (cancer setting)	3
2	F	30	Genetic counseling	8
3	F	27	Genetic counseling	4
4	M	47	Clinical genetics	28
5	F	45	Clinical genetics (cancer setting)	15
6	M	30	Oncology and surgery	5
7	F	30	Social work (cancer setting)	5
8	F	32	Clinical psychology (cancer setting)	7
9	M	32	Clinical genetics (cancer stetting)	12
10	F	55	Patient organization	7

### Interviews

The semi-structured interviews were focused on 3 main areas: (1) psychological and emotional needs, (2) medical and healthcare issues, and (3) individual and familial aspects. Interview guides are available in the supplementary materials. The interviews had a similar structure, with questions adapted for each category of participants; the professionals had an additional set of questions explicitly related to genetic counselling.

### Procedure

Most interviews were conducted face-to-face (by the first author) at the hospital or in participants’ workplace, with some interviews conducted on the telephone due to convenience (e.g., two patients were very keen to participate but lived outside the city and had difficulties travelling). On average, the interviews had a duration of approximately 30 min. Interviews were audio-recorded and transcribed verbatim. Before the interview, each participant had the opportunity to discuss the study at length and ask questions; all participants signed an informed consent form. The study was approved by the ethics committee of the Oncology Institute.

### Data analysis

**Fig. 1:**
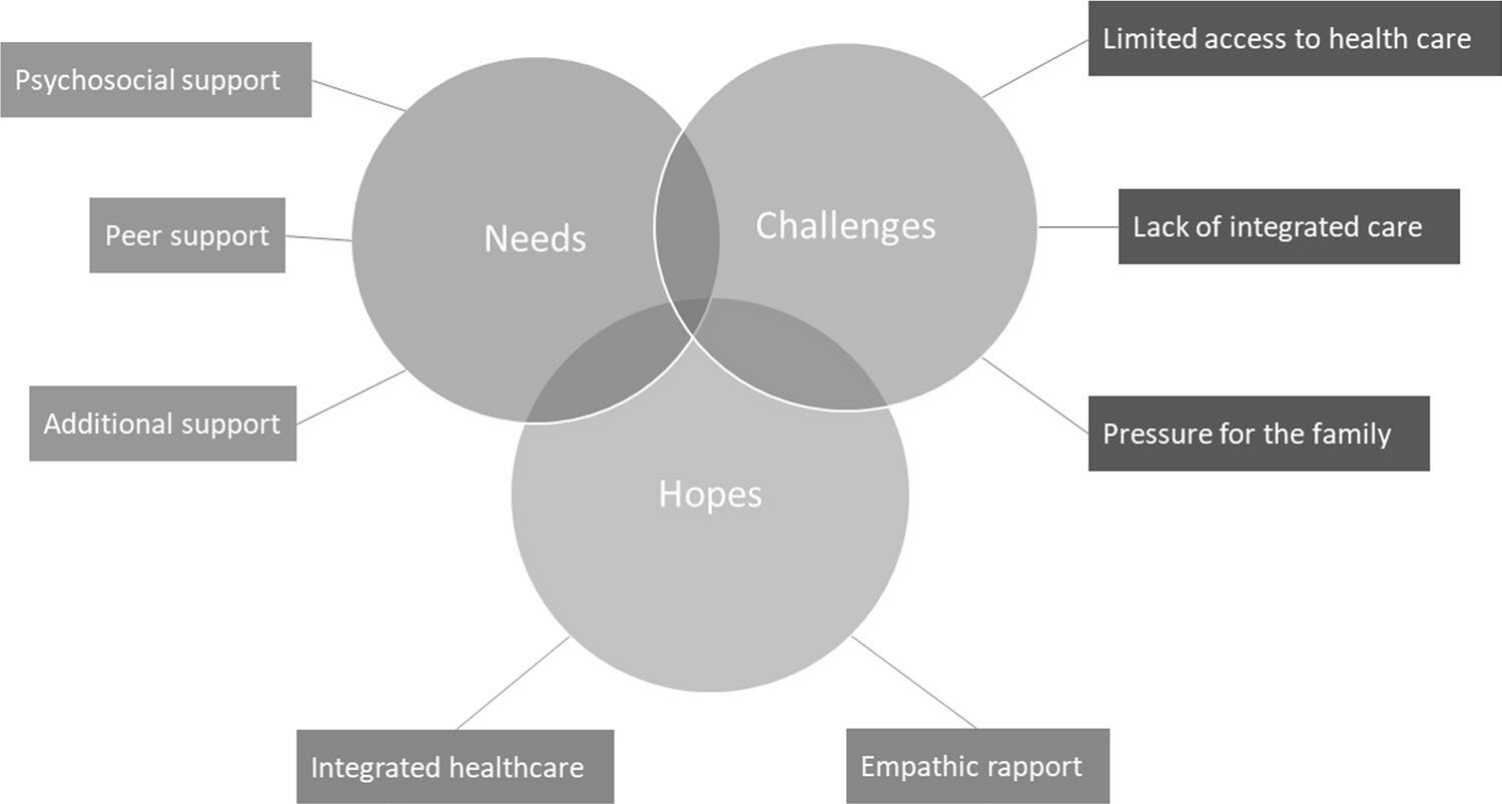
The thematic map

Thematic analysis was used to explore and identify the main themes in the interviews. Data analysis followed the procedure described by Braun and Clarke, 2006. In the first phase, the interviews were transcribed and observations about the data were noted. In the second phase, the interviews were coded in an inductive manner in order to extract relevant data. This phase was performed using a Microsoft Excel software based on the strategy proposed by Bree & Gerry, 2016. In the third phase, the codes were color coded and grouped based on color to facilitate theme searching. In the fourth phase, themes and sub-themes were identified, named and reviewed, and a thematic map was generated as shown in Fig. 1. The final step of the analysis was to select quotes that best capture the themes identified, the proposed research questions and overarching literature. The last interviews taken in each category brought only modest novelty to the pool of data already collected suggesting that data saturation was reached. This was also supported in the coding phase of the analysis when the emergent themes were supported by a significant number of codes (Fusch & Ness, 2015).

## Results

In total, 34 semi-structured interviews were conducted and analyzed. Following the thematic analysis, three major themes emerged: (1) Needs; (2) Challenges; (3) Hopes. Due to significant overlap and convergent ideas across all three categories of participants, the themes and subthemes are discussed together.

### Needs

The most salient theme across all interviews and categories of participants was the strong need for support, for both patients and families. Understanding the type of support patients and families need in cancer setting is essential for a functional and meaningful integration of genetic counselling services. Three sub-themes emerged here, each one describing different types of support needed.

#### Psychosocial support

Participants consistently mentioned the need to receive (i.e., patients, families) or provide (i.e., professionals) psychosocial support throughout the progress of their condition. Support is needed when coping with a cancer diagnosis and managing subsequent life changes, dealing with anxiety related to surgery and response to treatment, understanding implications for family members, dealing with intense negative emotions as well as the fear of being pitied. Patients also discussed needing support in managing the impact of the disease on family and couple relationships. Family members emphasized the need for a setting to discuss their own questions, concerns, or grief. Emotional and psychological support was most often seen in close connection with the medical care. Both patients and family members highlighted how struggling with negative emotions can interfere with understanding the medical information or adhering to treatment. Although there is clear consensus about the need for psychosocial support, professionals noted a reluctance of many patients to access psychological support services, mainly due to misconceptions or fear of being stigmatized for seeing a “mental health specialist”.

"*Maybe it would help to see a psychologist, or something. When you’re sitting here, in the hospital, there are times when you are on the ropes. You know, like in boxing, when you just can’t take it anymore. And sometimes doctors need to understand that. I have experienced it myself […]. Yes, emotionally… it’s hard.*" (Patient).

#### Peer support

Participants identified their peers and community as a valuable source of support. Patients often mentioned that they benefited from attending various support groups and family members said they would attend support groups themselves if they would find one tailored to their needs. Patients associations were described as “rescue lifelines” and several patients said they benefited from attending different meetings and conferences. Awareness campaigns for different types of cancer were described as being empowering. Some participants also acknowledged the value of fundraising campaigns either for individual cases or specific services, such as genetic testing. Maintaining an active professional life and connecting with work colleagues was also described as a valuable source of support. Professionals also discussed the benefits of peer support, or attending conferences; collaboration was mentioned particularly in the context of working or wanting to work in a multidisciplinary team. Several patients mentioned their desire to “give back” and share some of their experience, hoping that it might help others dealing with similar difficulties.

"*If you have people around you, if you have nice people in your hospital room… […], we go in the hospital park and we discuss a lot. I saw a lot of exhausted people and I said “C’mon people, why do you victimize yourselves so much? We are all here without various parts of our bodies because this is it… we all have these problems and we are all surviving, and we can be grateful for that.” And do you know what? After chats like that people started looking for us, for our room, because they felt that our group was emanating, how can I put it, health… and it helped them forget about the negative stuff.*" (Patient).

#### Additional support

Another sub-theme we identified was a distinct need for additional sources of support, in order to cope with long-term consequences and lifestyle changes following a cancer diagnosis. Some patients mentioned participating in research studies or their desire to do so. Some described using alternative medicine to help them cope with the side effects of the medical treatment. Several patients mentioned a specific need for “administrative” support, such as help to navigate medical services that are not reimbursed by the national insurance, their rights and available options, accessibility or just plain guidance in the hospital. Overall, most patients described an underlying need to focus more on their quality-of-life and enhance their personal autonomy. Spirituality and pastoral care were other essential sources of support for many participants. Patients and families consistently described the value of faith in coping with the diagnosis and some of them suggested religious groups could get involved in patient care.

"*Pff … Now I do not want to be that godly (laughs), but I think God has to help us. First of all, we need to have faith, trust… I don’t know how to tell you … Faith in God and trust in the doctors. Without doctors, there is nothing we can do, even if we pray.*" (Patient).

### Challenges

Early in the course of the interviews it became clear that the overarching context of the healthcare system will be essential in understanding the needs and challenges of the stakeholders involved. The three sub-themes that emerged here are detailed below.

#### Limited access to healthcare

This sub-theme highlights the difficulties some cancer families and professionals face with the national healthcare system. Whilst this may not be a challenge for individuals living in urban areas or with straightforward access to public or private hospitals, a number of individuals continue to struggle with access to adequate care. One of the most frequently mentioned challenges is the difficulty to access medical services due to long waiting lists, travelling arrangements, overcrowded hospitals and reduced number of staff. Some patients and family members also disapproved the paternalistic attitude some of the medical doctors continue to have. Regardless of the difficulties mentioned, patients and families expressed their empathy and gratitude towards the staff, and tried to remain hopeful for the future.

"*I think the hospital is too small for everybody coming here. The staff is not enough, they simply can’t cope. I think they are completely exhausted. Starting from the bottom and all the way up to the top. It’s too much, on everyone. I had a close look yesterday, when I came for my radiotherapy session, there were 687 admissions to the hospital. Just think about how many people are here!*" (Patient).

#### Lack of integrated care

This sub-theme highlighted the fact that integrated care for cancer patients is essentially lacking and there is a strong need to access (i.e., patients, family members) and provide (i.e., professionals) comprehensive, personalized care. The importance of multidisciplinary teams and the benefits of a good collaborative relationship between professionals and families were also highlighted. GPs were seen as having an essential connecting role within the team. Professionals also suggested the need for a tight collaboration between oncologists, surgeons, radiologists, nurses, geneticists, psychologists and genetic counsellors. The need for coherent, patient-centered public polices and good practice guidelines were also strongly emphasized.

"*There are difficulties when it comes to accessing ‘other’ services, like other than your own. There are no integrated services. So that’s the big problem! When it comes to genetics, you really feel it… I guess for other specialties too… *"(Professional).

"*I think the treatment would be more successful [In multidisciplinary settings]. Let me give you an example: in my experience, the patients I am seeing as part of a team have better outcomes than patients seen by myself or my colleagues alone. Individually we might miss something but as a team, we are better.*" (Professional).

#### Pressure for the family

Family members of individuals diagnosed with cancer face a number of challenges themselves. They act as advocates and information facilitators for the patient and, when the healthcare pathways are not straightforward, they often initiate the contact with various professionals and take initiative in “organizing” the care for their loved ones. Many family members also discussed the difficulties obtaining and understanding the medical information themselves. Often, they described their role as trying to persuade the affected individual to adhere to treatment or convincing other family members to undergo screening, without having a clear understanding of the implications that particular cancer diagnosis can have for the wider family.

"*The expectations from my family were very high… I had to support him [my dad] with everything, help with doctor appointments and what not. I am coming [at the hospital] almost every day. He needed me to stay overnight after surgery, he was confused for a few days and risked getting out of bed as he probably did not know where he was. You know, anesthesia and a bunch of other factors… age maybe. And I stayed every day and every night when it was necessary. Now I come to bring him comfort.*" (Family member).

### Hopes

The third theme encompasses the transformational impact of health communication and personalized approach. Across most interviews, participants shared their experience with health information in general and genetics in particular. Participants touched upon hope, mainly as a result of health communication; patients discussed feeling hopeless after getting information from unreliable sources and in a few instances, professionals discussed how health communication can instill hope. Two sub-themes are included here.

#### Integrated healthcare

Most participants mentioned the idea of a more personalized approach to their care, particularly in complex situations where a cancer family history is present. The need to better understand symptoms, causes, treatment options, risk factors for themselves or others, and screening options were mentioned repeatedly. Participants hoped for an easier and more integrated access to genetic counselling services. Interestingly, and unexpectedly, patients and family members often described genetic counselling without actually naming it, ideally facilitated by a trained professional, in a dedicated type of appointment.

"*I’d like to see changes in terms of communication, but I also understand that the doctors don’t have time to communicate, you know? I mean, they do their job in a very professional way. But there is no professional that has in the job description only this task, to communicate to the patient and the family how things are. And then you get this feeling of insecurity because you feel somehow misinformed, but it is not out of bad will, but out of the stiffness of the health care system. It is very difficult and inefficient. But if there was a way around this… I am talking about another kind of professional here… like in other areas, you have some kind of a spokesperson or something, you know?*" (Family member).

Patients and family members were generally aware when a history of cancer was present in the family and, in several instances, they questioned the inheritance pattern or the idea that cancer was in fact inherited. During the interviews, several myths related to the causes, genetics or inheritance of cancer became apparent such as inheritance only by males/females in the family. In one instance a patient described her surprise to find out that her adolescent son had an appointment with the GP to discuss his mother’s diagnosis and to understand more about his risks. In other instances, family members described increasing the frequency of screening or attending screening for the first time following a cancer diagnosis in the family, even if the additional care was not formally recommended by a doctor. Family members also described their difficulties in communicating sensitive medical information with the wider family or difficulties in obtaining informed consent for genetic testing. In this context, patients also discussed ethical aspects surrounding genetic testing such as “duty to warn” as a reason for testing and professionals mentioned difficult situations where they were asked to offer genetic testing to children and adolescents.

Professionals were clearly aware of genetic counselling and explicitly addressed it. Most of them mentioned the low awareness of genetic counselling and the unsystematic manner in which it continues to be offered to patients and families. Genetic counselling was described as having numerous benefits such as facilitating adaptation to cancer, understanding and managing risks, discussing the implications of genetic testing, facilitating decisions related to genetic testing or prophylactic interventions and providing emotional support throughout the entire process. Professionals, especially the clinicians and in some cases lab professionals, saw genetic counselling as a partial delegation of their own tasks (e.g., providing information and support), or a continuation of their role, beyond their expertise (e.g., discussing genetic testing in the case of oncologists). Genetic counselling was also described as a way to maintain hope and resilience in affected families.

Whilst most professionals stressed the need for genetic counselling, they also discussed several barriers in setting up this service, such as trained specialists, willingness to incorporate the logistics of a new service, insufficient funding of genetics and genetic testing, and the lack of a systemic interdisciplinary mindset. Some professionals proposed ways to address some of these challenges, such as training professionals to provide a basic level of genetic counselling only for conditions in their specialty (e.g., oncology, gynecology).

"*Often, it’s not the specialists who refers the patients [to genetic counselling], they come on their own – they might have seen a brochure, or they found out about us from the social media and so on. Other times they are indeed referred by the doctors but, honestly, let me tell you, it’s the same doctors every time. There are some who are so open and others who are probably totally uninterested or uninformed, I don’t know.*" (Professional).

#### Empathic rapport

This sub-theme is mainly focused on patients’ wish for a more “humane” interaction with the professionals and a more personalized communication throughout their care journey. The majority of participants described a great need for simple, plain language when discussing medical information. Some suggested professionals could use metaphors, visual aids or leaflets written in plain language to facilitate establishing a personal rapport. Many of the interviewed patients and family members suggested some professionals could benefit from additional training in communication. Using plain language was described as likely to increase the adherence to treatment, to improve the doctor-patient relationship and to help coping with cancer management, in general.

"*I think it would be important to have at least one genetic counsellor in every genetics department. At least one… if not an army of them! To have them talk to patients because they need someone who speaks their language, who empathizes with them, to really feel that empathy. And be a little bit more… more available than doctors. Not that doctors are not available but I think they would also really need a genetic counsellor.*" (Professional).

In their quest to understand the diagnosis, with everything it encompasses, the majority of the patients and family members said they tend to research medical information using various internet resources, and they almost always verify it with the medical team. Professionals often saw this as problematic due to misconceptions and truncated information they often have to subsequently address. Clearly, sharing the same language or narrative can enable a good rapport between professionals and families; equally, communication glitches can impact this relationship. Having said that, none of the professionals mentioned language or communication, either as a concern, challenge or a priority in particular.

"*The information I received was a bit too detailed, because I didn’t understand everything they were saying. I told him I didn’t understand and then he tried to explain it to me again. I don’t know, I guess he was very tired after the surgery, I don’t know… he was telling me words he knew, but I told him that I didn’t understand them… to take it a bit slowly because I had no idea what he was talking about. Also, I was incredibly nervous after the surgery. I guess I just wanted to talk a bit more in my own language, if possible.*" (Family member).

## Discussion

Our study was aimed at exploring the needs and challenges of patients, family members and professionals working in cancer settings, in a healthcare system where genetic counselling is not typically offered. We also wanted to see whether and to what extent genetic counselling is available or recommended. The findings uncovered key aspects of the current clinical practice in cancer settings, highlighting pressing needs and challenges amongst the interviewed stakeholders, as well as hopes and opportunities to bring genetic counselling services closer to the community.

The most prominent theme across all interviews and categories of participants was the need for support, for both patients and families. Patients and families were generally aware of a family history of cancer and often expressed concerns over adequately understanding the implications it had for their own diagnosis or the risks of other family members. Our findings also suggest that even some healthcare professionals may have difficulties assessing or interpreting a family history of cancer. In the relatively rare instances where professionals discuss hereditary cancers, most often they appear to concentrate on providing information, assessing risks, discussing screening or recommending testing. All three categories of participants discussed aspects surrounding informed consent, autonomy and confidentiality regarding genetic testing or other medical procedures. Several participants went on to discuss ethical concerns such as testing of children, equitable access to testing and the “duty to warn” family members about cancer risk. The wish to establish an empathic rapport, the value of personalized communication, and the interest and awareness for an ethical practice are all very much in line with the ethos of genetic counselling—facilitating and supporting patients’ autonomy and informed decisions.

The main challenges identified by most participants were limited access to healthcare and low availability of integrated care in cancer clinical settings. Patients and families mentioned having to access fragmented healthcare services which they have to navigate without much support. Most professionals echoed this challenge and tried to provide explanations or think of solutions. There was a clear consensus, and very much in line with the literature, that multidisciplinary team systems can address fragmentation because they promote good relationships and effective communication with team members, and include into decision-making processes patients’ choices, views and psychosocial factors (Soukup et al., 2018).

One particularly interesting finding was that most participants either clearly indicated or tentatively described the need to access genetic counselling services. Often, patients and family members described specific aspects of the genetic counselling process, without necessarily articulating how the service would look like or who the professional delivering that service should be. Although a large part of the recruited participants was unlikely to have a hereditary diagnosis, we systematically witnessed during the interviews myths and worries related to genetics and inheritance. Clarifying these myths and providing psychological support to individuals with increased anxiety related to cancer is an essential part of the genetic counselling process. That said, a potential bias of the sample included needs to be acknowledged.

There is substantial empirical evidence available to support the benefits of cancer genetic counselling, either when assessing it as a standalone service or when looking at separate components of the genetic counselling process, as our participants intuitively did. Discussing the family history can result in better healthcare outcomes, such as better screening attendance (Laiyemo et al., 2015) or uptake of cancer preventive measures (Metcalfe et al., 2008; van der Aa et al., 2015). Having a good understanding of cancer is has been shown to facilitate informed decisions (Martínez-Alonso et al., 2017). Receiving risk assessments has been positively associated with higher rates of screening for cancer (Rees et al., 2008). Having an appropriate understanding of genetic test results has been shown to be essential in facilitating a better adaptation to the diagnosis (Giri et al., 2018; Ersig et al., 2011; Taber et al., 2015). Genetic counselling can also effectively address misunderstandings regarding genetic testing (Borry et al., 2007) and facilitate family communication (Chivers Seymour et al., 2010).

When setting up a new service such as genetic counselling, in addition to advocating for the benefits it has been shown to have internationally and could have for other patients and families as well, one has to simultaneously acknowledge the local systemic difficulties and opportunities. Setting up a genetic counselling service in a developing healthcare system combined with the rapid developments in genetics and precision medicine can provide the opportunity to design and implement well thought service delivery models for genetic counselling (Stoll et al., 2018).

Our approach had a bottom-up perspective, aiming to identify the needs and perceived barriers of service users and providers, with a view to set up cancer genetic counselling services. Further research could collect complementary data from other stakeholders in order to better inform the parameters of new services, currently emerging but soon needing to be formally established. Clearly, policy and decision makers may have a different perspective; also, their input would likely have a broader viewpoint and that would undoubtedly bring a valuable input. Our findings could also be complemented with additional data from small towns or rural clinics, as regional experiences and views can significantly differ from large university hospitals.

The context of this study enables us to learn how genetic counselling services could be best tailored in order to address the challenges of a developing healthcare system. Locally, our study provides groundwork research for a more systematic approach aimed at integrating genetic counselling in clinical cancer settings. In the light of our results, a successful cancer genetic counselling service should actively seize the challenges and opportunities in the healthcare system to build an interdisciplinary and highly innovative service whilst being grounded in the local reality.

To conclude, our study explored the perceived barriers and opportunities of the main stakeholders in cancer settings in Romania, with a view to establishing a cancer genetic counselling service. Our findings highlighted the main needs, challenges and hopes the patients, family members and professionals have. Patients and family members mentioned needing genetic counselling services without actually naming them explicitly or even being aware that this type of service could in fact be available. Professionals, on the other hand, were very explicit about the need to integrate genetic counselling in the mainstream care for cancer patients. Whilst there are clearly many barriers when trying to set up a new service such as genetic counselling, particularly in a developing healthcare system, the often-unexpected opportunities to design and implement well adapted services are often easy to miss yet are undoubtedly paramount.
